# SingleCAnalyzer: Interactive Analysis of Single Cell RNA-Seq Data on the Cloud

**DOI:** 10.3389/fbinf.2022.793309

**Published:** 2022-05-23

**Authors:** Carlos Prieto, David Barrios, Angela Villaverde

**Affiliations:** Bioinformatics Service, Nucleus, University of Salamanca, Salamanca, Spain

**Keywords:** ScRNA-seq, data visualization, single cell, web server, data analysis

## Abstract

Single-cell RNA sequencing (scRNA-Seq) enables researchers to quantify the transcriptomes of individual cells. The capacity of researchers to perform this type of analysis has allowed researchers to undertake new scientific goals. The usefulness of scRNA-Seq has depended on the development of new computational biology methods, which have been designed to meeting challenges associated with scRNA-Seq analysis. However, the proper application of these computational methods requires extensive bioinformatics expertise. Otherwise, it is often difficult to obtain reliable and reproducible results. We have developed SingleCAnalyzer, a cloud platform that provides a means to perform full scRNA-Seq analysis from FASTQ within an easy-to-use and self-exploratory web interface. Its analysis pipeline includes the demultiplexing and alignment of FASTQ files, read trimming, sample quality control, feature selection, empty droplets detection, dimensional reduction, cellular type prediction, unsupervised clustering of cells, pseudotime/trajectory analysis, expression comparisons between groups, functional enrichment of differentially expressed genes and gene set expression analysis. Results are presented with interactive graphs, which provide exploratory and analytical features. SingleCAnalyzer is freely available at https://singleCAnalyzer.eu.

## Introduction

Single-cell RNA sequencing (scRNA-seq) has allowed for the quantification of RNA transcripts within individual cells. These assays allow researchers to explore cell-to-cell variability and meet new scientific goals. In the last few years, scRNA-seq has been applied, for example, to differentiate tumor cells from healthy ones, deconvolute immune cells, describe states of cell differentiation and development, and to identify rare populations of cells that cause disease (Haque et al., 2017). Although experimental scRNA-seq assays are becoming increasingly user-friendly, the analysis of sequencing data is complex. Data analysis requires the application of complex computational pipelines and data analysis methods that require bioinformatics expertise (Hwang et al., 2018). The interpretation of scRNA-seq results is strongly influenced by its analysis pipeline, and the incorrect application of methods could lead to conclusions that are incorrect. Since data analysis is complex and very important for correctly interpreting results, the development of analysis tools that produce reliable results and minimize the possibility of error is essential for enhancing the usefulness of scRNA-seq data.

Throughout the last 5 years, some software development projects have aimed to address the absence of software available for the analysis of scRNA-seq data (Guo et al., 2015; Gardeux et al., 2017; Kiselev et al., 2017; Lin et al., 2017; Perraudeau et al., 2017; Zhu et al., 2017; Scholz et al., 2018; Wagner and Yanai, 2018; Chen et al., 2019; Monier et al., 2019; Stuart et al., 2019). Designers of the projects have developed analysis pipelines that can be executed with R or Python function calls or with websites. Although the platforms have tremendous utility, they do possess some usability and functionality limitations that should be solved. For example, none of the applications are capable of analysing raw sequencing files (FASTQ), they do not allow for the interactive selection of groups and a few provide an integrated functional analysis of results (see Supplementary Table S1).

We have developed SingleCAnalyzer to provide a Web application server that performs a fully interactive and comprehensive analysis of scRNA-Seq data with two simple steps. It provides an integrated and interactive platform which is able to process sequencing files (FASTQ) and perform full scRNA-seq analyses and the functional analysis of results. It was implemented as a cloud analysis platform that can be executed without installing any software. SingleCAnalyzer facilitates the analysis of scRNA-seq data to non-experienced users and provides quick exploratory analyses to computational biologists.

## Results

### The SingleCAnalyzer Website

**FIGURE 1:**
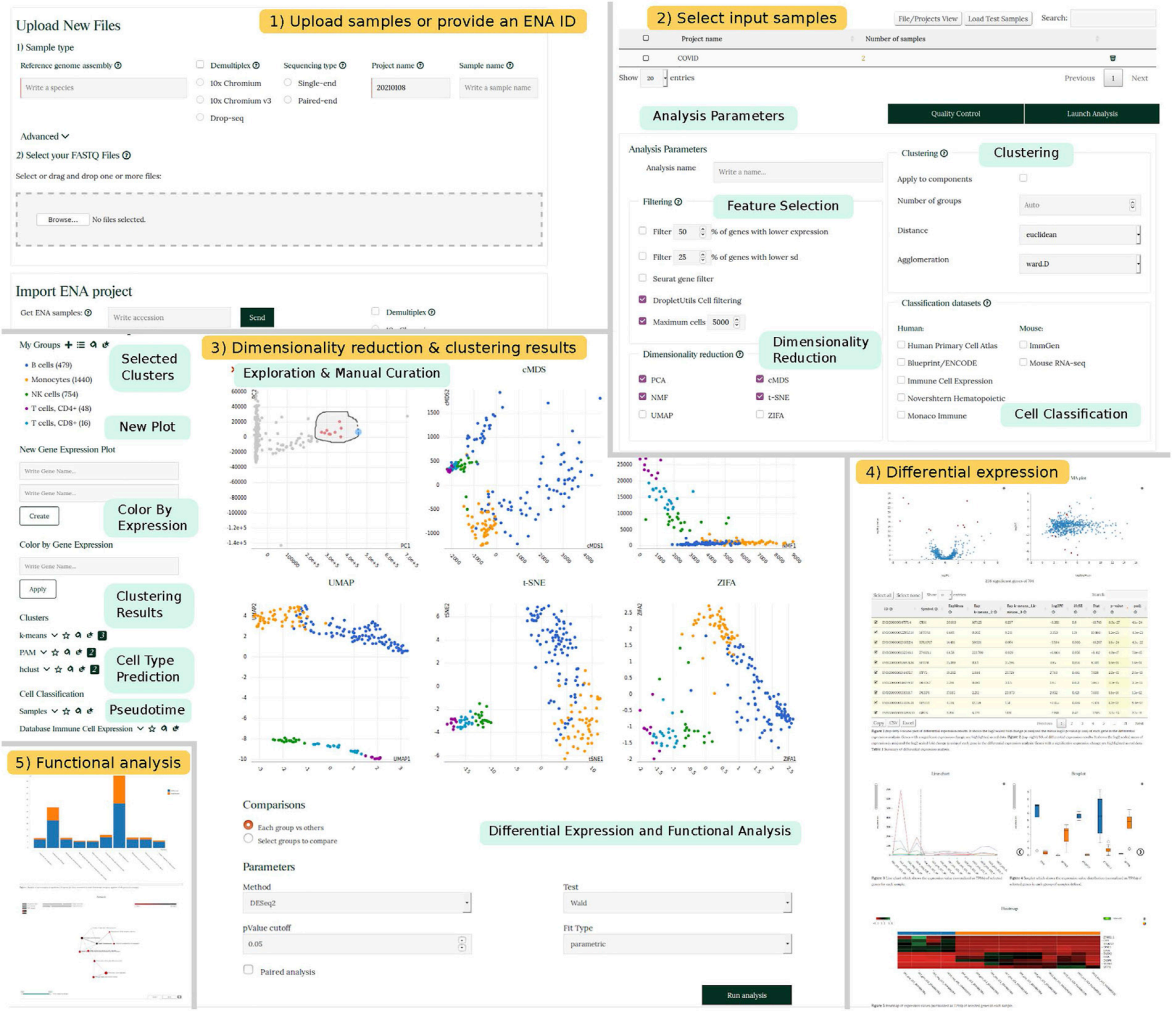
SingleCAnalyzer Workflow. Schematic representation showing SingleCAnalyzer workflow example. Panel 1 and 2 show the web interface for setting input files and parameters. Panel 3 shows an example of visualization of dimensionality reduction, cellular type classification, pseudotime analysis and clustering results. Panels 4 and 5 show selected sections of differential expression and functional analysis results.

The front-end of SingleCAnalyzer has been designed to provide a means to fully analyse scRNA-Seq data using the following two steps: 1) Setting input files and analysis parameters and 2) cluster determination and the execution of comparative analysis. In the first step, FASTQ/HDF5 files are uploaded or an ENA project identifier is provided by the user. Basic information regarding the species studied and type of sequencing performed, as well as optional parameters for the alignments of sequences can also be specified on the web. Once the files are uploaded, demultiplexed and aligned, users may perform further analysis including feature selection, empty droplet deletion, dimensional reduction, prediction of cellular type, analysis of trajectories/pseudotime and unsupervised clustering. These analyses can be performed and adjusted by selecting parameters in the ‘analysis parameters’ section (Figure 1).

Cluster determination and the execution of comparative analysis is accomplished through the website, which provides an interactive interface that allows the user to visualise cellular type prediction, pseudotime predictions or clustering results via six interconnected scatterplots generated using each dimensionality reduction technique. Point colour and type can be changed according to each analysis results. Users can also generate new representations of gene pairs and colour the points based on gene expression values. This interface specifies the most adequate aggrupation, cellular classification or time frame and is guided by the user’s knowledge regarding the samples studied. On the interface, the user can also launch a comparative analysis of all groups, or manually determine which groups should be compared. The comparative analysis includes an analysis of differentially expressed groups of genes, and the functional analysis of gene ontology categories and pathways.

Results are displayed in tabular form, which reveal the execution status of each computational process and provide a link to final results. These are provided as static reports and interactive web pages. Results regarding the quantification of gene expression values are provided with a table of quantification statistics and downloadable files that contain information for aligned reads regarding the number of reads generated per transcript and the number of transcripts per million (TPM). The quality control page descriptively reveals the distribution patterns of expression using box plots, reveals estimated numbers of expressed genes using a bar plot and represents the first two components of a PCA analysis. The clustering results page integrates dimensionality reduction, clustering, pseudotime and cellular classification results within self-explanatory interface which can also generate static reports that incorporate user modifications and launch comparisons between groups. Reports containing results are generated for each comparison, which include differential expression, functional enrichment and GSEA analysis. Differential expression results are summarised in a table which is linked to the following means to visualise data: MA plot, volcano plot, box plot, line chart and heatmap. Functional analyses are also summarised in tables and interactive visual means to represent data such as bar plots, networks and symmetric heatmaps are provided.

Supplementary Table S1 shows a comparison with 12 scRNA-Seq analysis platforms. The main features of the SingleCAnalyzer website are:

- scRNA-Seq analysis from raw FASTQ, HDF5 files or ENA project identifications
- Fully functional cloud platform that does not require the installation of software
- Semiautomated analysis which avoids the need for configuration using complex parameters
- User guided classification of cells within groups that is guided by interconnected graphs that integrate dimensional reduction, cellular type prediction, trajectory analysis and unsupervised clustering results
- Performs FASTQ processing, gene filtering, empty droplets detection, gene quantification, dimensionality reduction, unsupervised clustering, differential expression, functional overrepresentation and gene set expression analyses
- Straightforward presentation of results using interactive visual representations of data and provides a means to generate reports that are publication ready

### Analysis Pipeline

**FIGURE 2:**
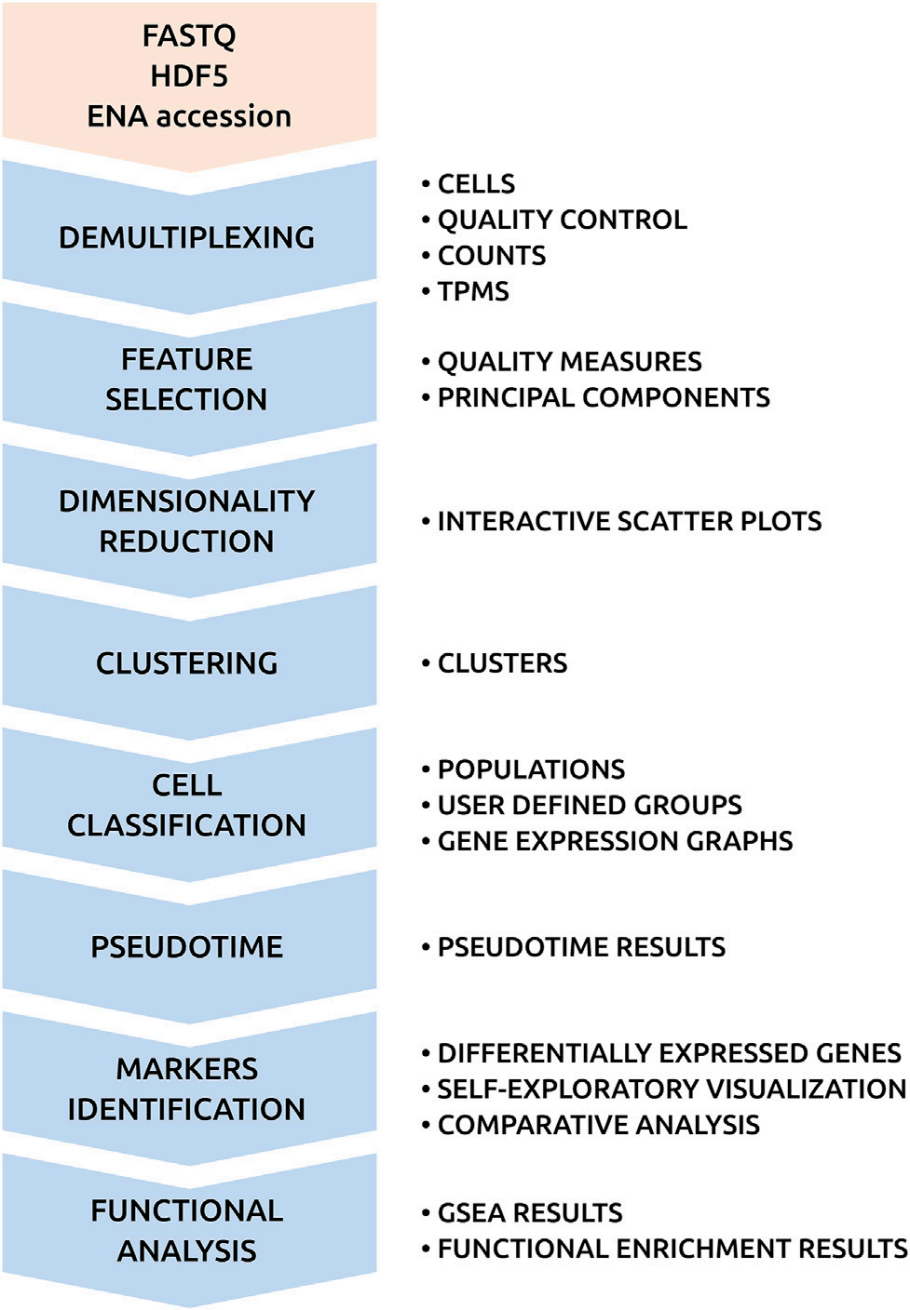
SingleCAnalyzer Pipeline. Chart of SingleCAnalyzer pipeline with computational processes and output results.

Figure 2 shows the analysis pipeline of SingleCAnalyzer. It integrates generally accepted tools used for the analysis of RNA-Seq data, which also perform well as computational resources. Supplementary Table S2 shows the computational time required to analyse nine scRNA-Seq public data sets. The complete analysis of 154 demultiplexed samples takes an average of 36 min, which allows for the real time analysis of low cell number scRNA-Seq experiments. The most time-consuming processes in the pipeline involves the upload, demultiplexing and alignment of samples, which are tasks that are performed in parallel. This parallelisation reduces the global analysis time by 57%, which makes the time requirement of our cloud infrastructure equal to virtual machine or local pipelines solutions. Moreover, SingleCAnalyzer does not store raw sequences or aligned files in order to avoid user disk space limitations, and the number of analysed samples of non-commercial cloud platforms.

The next steps of the pipeline include feature selection, empty droplets detection, dimensionality reduction, cellular type prediction, trajectory/pseudotime analysis and unsupervised clustering. SingleCAnalyzer applies gene filtering, which is based on user input parameters to avoid non-informative output, noise or drop out events. Afterward, six dimensionality reduction methods are applied to the data and samples are visualised using interactive scatter plots. Simultaneously, four unsupervised clustering algorithms are applied to produce nine possible clustering divisions for each method, a cellular type prediction method is executed for each training dataset, and a pseudotime analysis is performed (see methods). These cluster types can be mapped on interactive plots at the request of the user.

Based on the unsupervised or manually curated clusters produced, users can identify gene characteristics and the functions of each group by launching comparison analysis. This feature incorporates the differential expression analysis of groups and the functional analysis of gene ontologies and pathways. The analysis pipeline also processes quality control, clustering, differential expression and functional analysis results, and integrates them in an interactive and self-explanatory web interface.

SingleCAnalyzer was conceived as an agile project, and new scRNA-Seq analysis methods can be integrated within its analysis pipeline. Only generally accepted methods that have been demonstrated to generate reliable and reproducible results that require reasonable quantities of computational resources will be considered for addition to our cloud platform. The increasing development of computational methods will inspire the adaptation of the platform to meet the needs of researchers as scientific trends regarding scRNA-SEQ data analysis emerge.

### Interactive Visualization

Visualization is a key aspect on the interpretation of scRNA-Seq results (Cakir et al., 2020). Analysis pipelines performs scatter plots for the representation of dimensional reduction results where point colors represent clusters, cell types, gene expression or trajectory features of each cell (Kiselev et al., 2017; Lin et al., 2017; Stuart et al., 2019). These plots are adequate for publishing results, but not for explorative analyses. At present, new technologies based on JavaScript enable the generation of interactive graphs in a Web User Interface. They allow the connection between graphs and the use of HTML5 components which could control visualization aspects. SingleCAnalyzer adopts this technology to visualize information, interconnect graphs, show meta-information, calculate descriptive statistics, generate new graphs under user request and change the representation features interactively. SingleCAnalyzer includes six different graphical representations such as scatter plot, bar chart, heatmap, network, boxplot and density plot. These graphs are interactive, and the user can modify them by clicking on tables, html controls or other graphs.

The central result page is the representation of dimensional reduction and clustering of cells. It is composed by scatter plots where points represent cells, and the user can select the color and shape of points manually or by using clustering, cell population, pseudotime or gene expression results. The user can also explore group frequencies and define resulting groups based on meta-information or cell disposition on the graph. All graphs are interconnected, changes on graphical attributes or cell selections are synchronized on all displays. Cells can be located in all the graphs with a selection over one graph or by means of the locate samples menu. The application also allows the generation of new scatter plots which represent the expression of two genes in each cell.

Once the user defines the groups, he can launch a comparative expression analysis which results in two types of interactive reports. One is the differential expression report which are composed of interactive scatterplots, a boxplot, a line plot and a heatmap. All these graphs show information on mouse action and are connected with the table which summarizes the statistical analysis. They enable the comprehensive exploration of results and the query of information about expression changes of genes. The other report is the functional analysis which includes self-explanatory graphs such as bar plots, networks of terms and triangular heatmaps. Networks and heatmaps represent relations between gene sets which helps in the identification of related gene functions or pathways, while the bar plot shows the number of observed versus expected genes in each category.

Visualization features of SingleCAnalyzer enable the exploration and interpretation of results in an integrated platform which covers the main steps of scRNA-Seq analysis. The platform was presented and discussed at the VIZBI21 conference, where some improvements were suggested by attendants (VIZBI, 2022). Suggestions were focused on improving the usability of the platform and the adaptation of the analysis pipeline for their objectives. For example, an attendant required an adaptation for the analysis of RNA-Seq data which was developed and can be executed disabling the multiplexing process. SingleCAnalyzer is also distributed as a Docker machine and our graphical functions will be made public as R packages for its open use in analysis pipelines. All the representations performed with SingleCAnalyzer can be downloaded as graphical files ready for its inclusion in publications and analysis reports.

## Material and Methods

### Implementation

The SingleCAnalyzer website runs using LAMP architecture (Linux, Apache, MySQL and PHP). The front-end of the website was developed using PHP, HTML5, JavaScript, D3, JQuery, AJAX and CSS3. Its implementation was based on the RaNA-Seq project, which contains similar alignment, differential expression and functional analysis tools (Prieto and Barrios, 2019). The analysis pipeline can be executed by a task manager that runs the analysis processes using R, Python or Linux Bash Shell. It also balances the computational load on our high-performance computing cluster. The analysis pipeline integrates cutting-edge tools which rapidly and reliably analyse scRNA-Seq data. Figure 2 shows a flowchart of the pipeline used. We have optimised the analysis processes in our pipeline by harnessing computational clustering. Most of the tasks of analysis can be executed in real time. This optimisation has facilitated the development of an open and free cloud-based system.

### FASTQ Processing

Raw sequence files in FASTQ format can be demultiplexed with Alevin software (version 1.3.0) (Srivastava et al., 2019) or pre-processed using the Fastp tool (version 0.19.4) (Chen et al., 2018). Gene expression quantification of genes in the selected reference genome is performed using Alevin or Salmon software (Patro et al., 2017). The platform can be used to assess data generated from any organism. At present, we have downloaded the most popular genomes from Ensembl (164 genomes) and have incorporated their transcriptome indexes within our server (Cunningham et al., 2019). Quality control of samples is performed based on the alignment summary, descriptive statistics and the Alevin report of demultiplexed samples non-supervised clustering performed using AlevinQC package (version 1.4.0).

### Gene Filtering

Gene filters based on the quantification of gene expression, which reduce the noise and computational costs are available on SingleCAnalyzer. The current version can filter genes with the lowest levels of expression or standard deviations. We have also integrated the function ‘FindVariableFeatures’ within the Seurat package (version 3.2.2), which can identify variably genes by considering the strong relationship between variability and expression level (Stuart et al., 2019). Moreover, the user can also perform further dimensionality reduction and clustering processes by analysing the principal components obtained via principal component analysis (PCA). The optimum number of components used for the analyses can be determined using the *calc_npc* function of the CIDR package (version 0.1.5) (Lin et al., 2017). Empty droplets can be detected and removed with the application of the DropletUtils tool (version 1.8.0) (Lun et al., 2019).

### Dimensionality Reduction

Interactive visualisation of samples in scatter plots requires a dimensionality reduction process, which is performed using the following methods: 1) PCA, which is generated with the *prcomp* function of the *stats* R package (version 4.0.3); 2) Classic multidimensional scaling (cMDS), which is performed with the *cmdscale* function of the *stats* R package using camberra as distance method; 3) Nonmetric multidimensional scaling (isoMDS), which is performed using the *isoMDS function of the MASS* R package (version 7.3); 4) t-distributed stochastic neighbor embedding (t-SNE), which is performed using the *Rtsne* function of the *Rtsne* R package (version 0.15); 5) Uniform manifold approximation and projection (UMAP), which is performed using the *umap* function of the *uwot* R package (version 0.1.9); 6) and Non-negative matrix factorisation (NMF), which is performed using the *nnmf* function of the *NNLM* package (version 0.4.3)*.* Collectively, application of these methods provides users with a multi-perspective assessment of the relationships between data.

### Unsupervised Clustering

Determination of clusters within the interactive web interface is supported by the results provided by unsupervised clustering methods. At present, SingleCAnalyzer applies the following unsupervised clustering methods: 1) k-means, which is computed using the *kmeans* function of the *stats* R package (with iter_max = 15); 2) partition around medoids (PAM), which is computed using the *pam* function of the *cluster* R package (version 2.1.0); 3) hierarchical clustering, which is performed using the *hclust* function of the *stats* R package; 4) leiden clustering and pseudotime analysis, which is performed using Monocle3 R package (version 0.2.3) (Qiu et al., 2017)*.* The user can specify input parameters such as the desired number of groups, the distance metric used by pam and hclust functions and the agglomeration parameter of hclust.

### Pseudotime Analysis

Trajectory and pseudotime analyses are performed using the Monocle3 R package (Qiu et al., 2017). It calculates possible trajectories between leiden clusters over the UMAP projection. The pipeline calculates the pseudotime prediction for each cluster centroid and a scale colour which represent the time is applied over the points when an origin cluster is selected. The function preprocess_cds uses PCA or LSI output based on user options with the following parameters: norm_method = log and scaling = true. The function reduce_dimension uses the following parameters: max_components = 2, reduction_method = UMAP, umap. metric = cosine, umap. min_dist = 0.1, umap. n_neighbors = 15L, umap. nn_method = annoy. The function cluster_cells uses the following parameters: k = 20, cluster_method = Leiden, nunm_iter = 2, partition_qval = 0.05. The function learn_graph uses use_partitium and close_loop as true.

### Comparison Between Clusters

Groups of samples can be compared by applying different methods to assess differential expression. Reviews of the use of methods have concluded that no single method outperforms the others under all circumstances, and suggest that it is necessary to determine the optimal method or pipeline for each analysis performed (Seyednasrollah et al., 2013; Soneson and Delorenzi, 2013). However, researchers have acknowledged that DESeq2 (version 1.28.1) (Love et al., 2014), EdgeR (version 3.30.3) (Robinson et al., 2010) and limma (version 3.44.3) (Law et al., 2014) are the most widely used methods and consistently performed well when their reliability was assessed. We have integrated all of the methods within a SingleCAnalyzer that can be adjusted to apply customised parameters to individual tests.

SingleCAnalyzer performs a functional enrichment analysis and a gene set enrichment analysis (GSEA) for each comparison result. The enrichment analysis is performed with the R package GOseq (version 1.40) (Young et al., 2010) and the GSEA is performed with the R package fgsea (version 1.14) (Korotkevich et al., 2016). Functional annotation database used by these methods was downloaded from the NCBI BioSystems repository (Geer et al., 2009). Resulting graphs are generated with the package RJSplot (version 2.6) (Barrios and Prieto, 2018).

### Data Management

Analyses can be launched as anonymous or registered users. Anonymous accounts are regularly deleted, and registered users can require the cancellation of their account. Data of registered users are protected by their personal password which is encrypted on our system. Users can freely download or delete their processed data and analysis results without any limitation. Raw data files uploaded by users (FASTQ, HDF5) are deleted once they are processed. This deletion avoids storage limitations and the presence of sequences in our system.

## Discussion

Single-cell platforms provide computational methods which enable the transformation of sequences into expression values of genes in each cell (Zheng et al., 2017; Shum et al., 2019). Further steps can be performed by the application of bioinformatics methods which are available on code repositories or analysis servers. These methods are connected in series to compose an analysis workflow. The development of pipelines is a complex work which involves the installation, test, setting up and integration of computational methods. In addition, full processing of scRNA-Seq data requires an intensive computational processing and the knowledge of programming languages for the execution of the pipeline. On the other hand, cloud servers are designed to avoid the development and execution of pipelines by the analysts, but its use also implies limitations such as additional data uploading time, uncertain server loads and limited customization of the analysis. Previous works have provided web servers for the analysis of scRNA-Seq data from a matrix with gene counts of cells (Gardeux et al., 2017; Zhu et al., 2017; Scholz et al., 2018; Chen et al., 2019; Monier et al., 2019). In this work we have developed the first cloud server which allow a complete analysis from sequences to pathways in a fully integrated platform. It was possible with the integration of low computational cost methods for the demultiplexing and quantification of reads which supports Drop-seq and 10x Chromium single-cell protocols (Srivastava et al., 2019).

Another approach for the analysis of scRNASeq sequences is the use of workflow management systems. A popular option is Galaxy which offers a web-based system for the pipeline construction and the execution of bioinformatic analyses (Jalili et al., 2021). A recent study has presented Galaxy workflows for the analysis of scRNASeq data (Moreno et al., 2021). One of the workflows allows the uploading of FASTQ files for processing into an annotated cell matrix with Alevin. Then, post processing is done with Scanpy (Wolf et al., 2018) and the interactive visualization with the UCSC CellBrowser (Speir et al., 2021). This workflow has similar limitations to cloud solutions, as customization and uploading time, and requires of a computational cluster account and training about Galaxy workflows. Regarding the integration of results, the application of standard visualization tools avoids the creation of custom interfaces which integrate different nature of results, and the execution of new analysis based on the user interaction with the graph cannot be performed.

Visualization is a key aspect on the interpretation of scRNA-Seq results (Cakir et al., 2020). An adequate and interactive representation facilitates the correct classification and characterization of cells. This issue has been extensively approached by analysis techniques of cytometry and visualization methods have been adapted to the specific characteristics of single-cell such as the lower number of cells and the increment on the number of variables (transcripts/proteins). Two dimensional plots have been traditionally used for the representation of fluorescent makers on Cytometry. At present, flow cytometry panels can include dozens of makers and its representation as scatterplots are performed by a dimensional reduction technique. Similar strategy is followed for single cell visualization, but the lower number of cells allows its representation with web-based technologies which avoids software installation and platform dependencies. SingleCAnalyzer has developed its graphical interface with D3 and JavaScript technologies which allows the user-graph interaction on a Web browser. This solution has efficiently tested for the representation of 6,000 cells on six simultaneous scatterplots and allows a full interaction with clustering, cell classification, transcript quantification and cell trajectory results. Regarding the differential expression interface, it can handle 60,000 transcripts and perform six interconnected representations (MA-plot, volcano plot, scatterplot, boxplot and heatmap) on user interaction. The scalability of the platform will depend on the optimization of Web Browsers in the storage, representation and processing of interactive HTML Canvas and Scalable Vector Graphics. Current browsers have memory management and multiprocessing limitations. However, these technologies are becoming popular, and browsers are adapting their rendering engines for improving their performace (e.g. RenderingNG technology of chrome).

Future implementations of SingleCAnalyzer will be directed to the integration of novel analysis methods for scRNA-Seq and to the compatibility with new platforms and experimental protocols. At present, we provide semi-automated analysis of scRNA-Seq data on the cloud with analytical and interactive graphs, which enable the comprehensive analysis of results. It is freely available for scientists to explore the potential of their scRNASeq studies running quick analysis on an easy-to-use interface.

## Data Availability

Publicly available datasets were analyzed in this study. This data can be found here: https://singlecanalyzer.eu.
