# Supplementary material for: SingleCAnalyzer: Interactive Analysis of Single Cell RNA-Seq Data on the Cloud
Source: Front Bioinform. 2022 May 23;2:793309. doi: 10.3389/fbinf.2022.793309 (PMC9580930; doi:10.3389/fbinf.2022.793309)
Supplement: Supplementary file 1 [file DataSheet1.PDF]

Name	Implementation	FASTQ Processing	Gene Filtering	Dimensionality Reduction	Clustering	Interactive Clustering	Differential Expression	Functional Analysis	Cell populations / pseudotime	Comprehensive Reports	Setting of Parameters	Spatial Plots	PMID
**SC3**	R	No	Yes	Yes	Yes	No	Yes	No	Yes	No	Yes	No	28346451
**ASAP**	Web/Cloud	No	Yes	Yes	Yes	Yes	Yes	Yes	Yes	No	No	No	32449934
**Granatum**	Shiny web server	No	Yes	Yes	Yes	No	Yes	Yes	Yes	No	No	No	29202807
**SEURAT**	R	No	Yes	Yes	Yes	No	Yes	No	Yes	No	Yes	Yes	31178118
**MOANA**	Python	No	Yes	Yes	Yes	No	Yes	No	Yes	No	No	No	NA
**STREAM**	Web/Cloud	No	Yes	Yes	Yes	No	No	No	No	No	No	No	31015418
**SINCERA**	R	No	Yes	No	Yes	No	Yes	Yes	Yes	No	Yes	No	26600239
**CIDR**	R	No	Yes	Yes	Yes	No	No	No	No	No	Yes	No	28351406
**iCellR**	R	No	Yes	Yes	Yes	No	Yes	Yes	Yes	No	Yes	Yes	NA
**IRIS - EDA**	Shiny web server	No	Yes	Yes	Yes	No	Yes	Yes	No	No	No	No	30763315
**Biocond. workflow**	R	No	Yes	Yes	Yes	No	Yes	No	No	No	Yes	No	28868140
**FASTGenomics**	Web/Cloud	Yes	Yes	Yes	Yes	No	Yes	No	No	No	No	No	NA
**SingleCAnalyzer**	Web/Cloud	Yes	Yes	Yes	Yes	Yes	Yes	Yes	Yes	Yes	No	No	NA

Supplementary table S1. Comparison of pipelines, web servers and cloud platforms for the analysis of scRNA-Seq data.

Project Identifier	Organism	Nº Samples	Nº Fragments	Bases x Frag.	Uploading	Quantification	Counts	Clustering	DE	GSEA	GOSEQ	Total
**PRJNA229411**	Mus musculus	201	484268670	2x101	00h 24m 19s	00h 26m 41s	00h 00m 06s	00h 00m 12s	00h 06m 00s	00h 00m 38s	00h 00m 24s	**00h 33m 37s**
**PRJNA481319**	Homo sapiens	135	3193027317	66	00h 39m 39s	00h 43m 37s	00h 00m 06s	00h 00m 05s	00h 01m 32s	00h 00m 47s	00h 00m 27s	**00h 46m 07s**
**PRJNA314949**	Homo sapiens	384	837257090	2x125	00h 37m 31s	01h 27m 34s	00h 00m 11s	00h 00m 25s	00h 28m 17s	00h 00m 38s	00h 00m 34s	**01h 57m 05s**
**PRJNA296119**	Homo sapiens	121	502602411	2x100	00h 20m 22s	00h 26m 13s	00h 00m 06s	00h 00m 07s	00h 01m 44s	00h 00m 30s	00h 00m 17s	**00h 28m 40s**
**PRJNA286698**	Mus musculus	148	382295820	90	00h 04m 56s	00h 10m 15s	00h 00m 05s	00h 00m 07s	00h 05m 06s	00h 00m 35s	00h 00m 15s	**00h 16m 08s**
**PRJNA573109**	Homo sapiens	14	1176159212	58	00h 09m 23s	00h 11m 54s	00h 00m 02s	00h 00m 03s	00h 00m 21s	00h 00m 46s	00h 00m 39s	**00h 13m 06s**
**PRJNA503340**	Homo sapiens	72	543308382	50	00h 06m 36s	00h 08m 02s	00h 00m 04s	00h 00m 02s	00h 00m 28s	00h 00m 34s	00h 00m 25s	**00h 09m 10s**
**PRJNA558893**	Homo sapiens	66	1825947716	30+70	00h 27m 35s	00h 38m 07s	00h 00m 05s	00h 00m 03s	00h 00m 49s	00h 00m 40s	00h 00m 30s	**00h 39m 44s**
**PRJNA393431**	Danio rerio	246	272727507	2x38	00h 02m 42s	00h 08m 43s	00h 00m 06s	00h 00m 10s	00h 12m 04s	00h 00m 32s	00h 00m 16s	**00h 21m 35s**

Supplementary Table S2. Execution time of each process in the pipeline of SingleCAnalyzer. This test was executed on an Intel Xeon Gold 6146 processor (3.2 Ghz) with 256GB of RAM.
